# Legalization of Marijuana: Unraveling Quandaries for the Addiction Professional

**DOI:** 10.3389/fpsyt.2013.00050

**Published:** 2013-05-31

**Authors:** Roger A. Roffman

**Affiliations:** ^1^School of Social Work, University of Washington, Seattle, WA, USA

For more than four decades, a movement to shift cannabis control policy away from prohibition has gained momentum in such countries as Australia, Netherlands, Portugal, Spain, and the United States (Caulkins et al., 2012, pp. 207–224). When the issue is debated, researchers, and clinicians in the addictive behaviors field may face a difficult choice when asked where they stand with reference to making legal the retail sale of marijuana to adults. It is clear that there are health and behavioral risks to marijuana use. But, do those risks in and of themselves close the door to considering alternative policies? Or, to frame the issue somewhat differently, does prohibition's track record in protecting public health and safety justify its continuance when considered alongside one or another non-prohibition policy?

These questions are posed with increasing frequency as public attitudes in the U.S. concerning legalizing marijuana become more accepting. In 2011 the Gallup Poll found 50% of Americans saying marijuana use should be legal (Gallup, 2011). Then, in 2013 a Pew Research Center poll found that 52% favored legalization, a 10 point increase since 2010 (Pew Research Center, 2013). These indications of greater tolerance of marijuana appear to mirror a populist push back among voters and legislators in a number of states, a willingness to carve out exceptions to complete prohibition, the stance still held by the federal government.

The U.S. is a signatory to the 1961 Single Convention on Narcotic Drugs which requires all nations to prohibit both the production and use of marijuana for all purposes whether medical or non-medical (Single Convention, 1961). The subsequent 1988 Convention further requires each country to enact criminal penalties for the production, distribution, possession, or purchase of marijuana (United Nations Convention, 1988). Nonetheless, in the past 15 years, 18 states and the District of Columbia have adopted medical marijuana laws which essentially bypass the drug's Schedule 1 classification under the Controlled Substances Act, the national implementing legislation of the Single Convention. Its placement in Schedule 1 is based on the premises that marijuana has a high potential for abuse, has no currently accepted medical use, and has a lack of accepted safety for use under medical supervision. These treaties and laws notwithstanding, in 2009 the Obama administration made an accommodation to those states that had approved medical marijuana legislation. Individuals who were in compliance with those state laws would not be subjected to federal prosecution (Caulkins et al., 2012, p. 191). Then, in the months following the November 2012 election when Washington and Colorado voters approved a legal regulated marijuana market, the Obama administration remained essentially silent as to whether it would act to prevent those two states from implementing their laws’ provisions. The position taken by federal officials in the intervening months has been that marijuana will remain illegal under federal law.

Thus, the pendulum appears to be swinging further away from the full prohibition end of the policy continuum. Advocates for policy reform underscore the substantial adverse consequences of prohibition for society. First, a large black market largely nullifies efforts to prevent ready access to marijuana, with billions of dollars in profits enriching gangs and cartels and fueling egregiously high rates of violence and murder. The illicit marijuana that some 30 million Americans consume each year is therefore not subjected to regulations that might require accurate labeling of potency and cannabinoid ratios, testing to assure non-contamination, and limiting sales to adults. The burden on public coffers from the operational costs of the criminal justice system adds to the list. Then there's the question of social justice, with advocates emphasizing the dire consequences, many enduring for decades after the commission of the offense, experienced by an individual who is treated as a criminal for having possessed marijuana, e.g., loss of employment, loss of housing, loss of voting rights, loss of federal financial aid for college, seizure and forfeiture of property, termination of child visitation rights, and deportation for legal immigrants. The evidence of major racial inequities in how marijuana laws are actually enforced adds to the specter of injustice. Finally, another contributing factor is the prospect of billions in new tax revenues being generated annually (Miron and Waldock, 2010).

Yet, when the prospect of legalizing marijuana is raised as an alternative societal approach, colleagues in the addictions field often voice understandable apprehension about possible outcomes. From what I have learned as a marijuana dependence behavioral intervention researcher and addictions therapist, those concerns also trouble me. First, legalization likely will convey an erroneous message that marijuana use has no risks, and with that belief, attitudes will likely change and the prevalence of use, adverse consequences as well, may rise. Those who have been protected from harm because of their anti-drug attitudes and the stigma attached to marijuana use, due in part to its illegality, will lose that protection. Second, it can be expected to increase youth access to marijuana through diversion from legal outlets and by young people accessing their parents’ or older friends’ supply. Third, a legal market will attract entrepreneurs, a new industry will rapidly grow, and profit motives will fuel strong opposition to efforts to limit advertising, to selling high potency marijuana that may increase mental health risks, to labeling products to warn of adverse effects, to limiting where sales may take place and the density of sales outlets, and so on. Fourth, societal costs due to alcohol and tobacco morbidity and mortality, as well as traffic accidents and fatalities associated with driving under the influence, suggest a hefty burden taxpayers may carry once marijuana joins them as a legal commodity. In sum, in responding to the rationale for legalization, addictions professionals express concerns about its possible adverse impact on those who are vulnerable to abuse or dependence, with a particular emphasis on youth.

But, might the public and those who are vulnerable to harm be more effectively served were marijuana to be legalized? I've come to the conclusion that a closely regulated model of legalization that incorporates a true public health approach to addressing health and safety, an approach that stands a chance of preventing the ominous outcomes feared by addictions specialists, deserves consideration.

The measure approved by voters in the state of Washington is just such a model (Wash, 2013. Laws c. 3). It includes the following provisions, all of which will be funded by excise tax and license fee revenues generated by the legal marijuana market.

(1) Empowers an agency of state government to write and enforce regulations concerning growing and selling marijuana and marijuana-infused products such as confections. The word legalization conjures up a variety of possibilities, with one end of the continuum being solely the complete repeal of all laws prohibiting growing, possessing, and selling. In contrast, Washington's law calls for a tight regulation model. It mandates an agency of state government, the Washington State Liquor Control Board, with the responsibility to write and enforce regulations concerning such matters as the criteria applicants must meet in order to qualify for licenses to legally grow, process, or sell marijuana, the location and density of marijuana sales outlets, and limits to advertising. Washington's marijuana legalization law specifies that sales may only take place in stand-alone stores, a provision designed to minimize the marketing inherent in products being visible on the shelves of grocery or convenience stores.
(2) Implements both universal and targeted science-based public education. Misinformation concerning marijuana's risks is considerable. Too many young people underestimate the adverse consequences associated with regular use of marijuana by people in their age group (Johnston et al., 2012).
  The Washington law mandates several state agencies with the responsibility of educating the public through the dissemination of accurate information about marijuana and allocates earmarked funding for this purpose. Ideally, in carrying out this mandate these agencies will design their efforts to convey messages using empirically supported public education methodologies and tailor the delivery of those messages for specific population subgroups.
(3) Funds communities to mount science-based marijuana prevention programs. Much has been learned about what works in preventing young people from using drugs (National Institute on Drug Abuse, 2003), although funding limitations have considerably restricted the extent to which empirically supported prevention programming is delivered (Ringwalt et al., 2002). Earmarked funding to Washington's behavioral health and recovery agency will be devoted to implementing evidence-based primary and secondary prevention programs targeting middle school and high school-age students.
(4) Makes available empirically supported marijuana dependence counseling. A portion of the legal marijuana market tax revenues will be allocated to local health departments and/or community agencies to implement treatment interventions. Additionally, a marijuana use public health hotline will be established for the purpose of providing treatment referrals and delivering research-based harm reduction services. As is the case with empirically supported prevention, the diffusion of effective substance abuse treatment protocols remains quite limited (Garner, 2009).
(5) Evaluates the extent to which marijuana-related behaviors, adverse consequences, attitudes, and beliefs change over time, and uses these data to fine-tune the legal market's regulations. A key element in this new law is devoting funding to research activities designed to evaluate the law's impact. First, earmarked funding will support several state agencies in a collaborative biennial effort to survey students, with the goal of assessing such variables as students’ use of alcohol, tobacco, and other drugs; academic achievement; age of drug use initiation; antisocial attitudes and behaviors; community norms; family management and conflict; parental attitudes concerning drug use; and perceived risks of marijuana.

Second, funding will be allocated to an independent state agency that conducts policy-focused research. Periodic reports will be required from this agency over a 20 year period. The evaluation is to focus on public health, public safety, economic impacts of the law's implementation in the public and private sectors, and impacts from the activities funded by the law's education, prevention, treatment, and research provisions.

## Conclusion

Washington's approach to regulating marijuana undoubtedly will require fine-tuning in the years following its full implementation, a process which will unfold over time. It's anticipated that licensed retail sales outlets will first open sometime in the spring of 2014. Then, as excise taxes and license fees from the fully implemented legal marijuana system are collected by the state, the earmarked funding for the public health components of this new policy will begin to flow to the various state agencies. In essence, it will most likely be late 2014 before all of the new law's provisions have begun to be operational.

Because one component of this new policy is to fund impact evaluation from multiple sources over time, state government will have data to inform future marijuana control policies. In serving as a laboratory of democracy, Washington will have the opportunity to test a model of legalization that may point the way to more effective protection of both the general public and vulnerable populations than has been the case under prohibition (Hawken et al., 2013).

Nowhere in the United States or elsewhere has this model of legalization been adopted, thus offering little precedence on which to base projections. However, I believe that prohibition's track record in protecting public health and public safety has been seriously deficient. Moreover, inequities in prohibition's implementation make evident it has been fundamentally flawed in terms of social justice.

When the evaluation data begin to become available over the coming years, among the outcomes I hope to see, in contrast with what we have witnessed prior to legalization, are: fewer young people initiating marijuana use prior to age 21, fewer students struggling with school performance as a consequence of marijuana use, a smaller percentage of users becoming marijuana dependent, more of those who become dependent receiving effective treatment, fewer traffic accidents in which marijuana smoking is a contributing factor, and more accurate knowledge held by the public concerning marijuana's effects on health and behavior.

As other states consider establishing a regulated marijuana market, the Washington state model deserves a careful look.
